# Editorial Comment: Presentation delay, misdiagnosis, inter-hospital transfer times and surgical outcomes in testicular torsion: analysis of statewide case series from central Brazil

**DOI:** 10.1590/S1677-5538.IBJU.2019.0660.1

**Published:** 2020-09-02

**Authors:** Daniel Sá Rego Hampl

**Affiliations:** 1 Serviço de Urologia Hospital Municipal, Souza Aguiar Rio de JaneiroRJ Brasil Serviço de Urologia, Hospital Municipal, Souza Aguiar, Rio de Janeiro, RJ, Brasil

## COMMENT

Testicular torsion is a urological emergency related to spermatic cord torsion, leading to a compartment syndrome inside the rigid tunica albuginea and severe local ischemia. Time lapse from symptoms to surgery is the only predictor factor related to greater salvage rates. Deleterious impacts to the endocrine function and the sensitive spermatogenesis process are seen after only 2 hours of ischemia ( 1 ).

Delayed surgical approach is related not only to the twisted organ loss but also to unpredictable implications for the contralateral testicle. The inflammatory syndrome causes recruitment of a wave of oxidant agents and anti-sperm antibodies, which can harm the remaining testicle ( 2 ).

There are many uncertainties related to management of testicular torsion, such as ascertainment of organ viability, definition of drugs that can prevent reperfusion syndrome, choice of tunica vaginalis patch, tunica albuginea incision, process to monitor the saved organ and determination of the ideal time for reconstruction with prosthesis implant ( 3 ).

On the other hand, there is a categorical statement that must be followed to improve salvage rates: surgery must be performed as soon as possible. To guarantee prompt surgical treatment, surgeons are allowed to perform scrotal exploration when there is a clear clinical suspicion with no need for complimentary exams.

In clinical practice, we can identify multiple reasons for delayed treatments. While the long time lapse from symptoms to first medical assessment depends on the patient’s awareness of the disease ( 4 ), subsequent delays, such as long inter-hospital transfer time, are healthcare system issues ( 5 ).

There is misuse of the concept that salvage rates are greater when surgery is performed within six hours by physicians that routinely disparage urgency when patients’ first consultation occurs after that period. We know that testicular survival after a torsion event can be expected after a longer period, so this information should discourage therapeutic nihilism when torsion pain has lasted longer than six hours ( 6 ).

In addition, in recent years the urologic specialty has become consolidated, ultrasound machines have become available in most emergency units, and the number of malpractice suits against physicians has grown. Those factors could be reasons why primary units are missing clinical diagnosis (waiting for confirmatory ultrasound) and why general surgeons are avoiding the surgery.

In the study, testicles were only salvaged in 46.1% of cases. Despite the retrospective method, the authors were able to identify and clearly show some of the most important factors of the Brazilian healthcare system that could be improved to achieve better testicular torsion treatment outcome ( 7 ).

The authors could point out that ultrasound was more often requested by primary and secondary units, which led to delayed treatment. Salvage rates were greater when first clinical examination took place in tertiary units that did not use radiologic exams frequently.

All people involved to public health policymaking in Brazil should consider those findings, to change the way of dealing with this condition, whose treatment can be mutilating and full of unpredictable impacts on men’s health.
